# 3D Printing in Medicine: an introductory message from the Editor-in-Chief

**DOI:** 10.1186/s41205-015-0001-5

**Published:** 2015-11-27

**Authors:** Frank J. Rybicki

**Affiliations:** The Ottawa Hospital Research Institute and the Department of Radiology, the University of Ottawa Faculty of Medicine, 735 Parkdale Avenue, Ottawa, ON K1Y 4E9 Canada

Personalized medicine and precision medicine are easier to conceptualize than define, and implementation can be even more challenging. 3D printing has intersected medicine to enable both. Personalized medicine is now delivered by “clinical modelers”, impassioned investigators are caretakers who model disease with 3D printing to define pathology, plan intervention, and treat patients.

Creating, manipulating, and printing Standard Tessellation Language (STL) files is challenging; generating a hand-held model from a CT scan is harder than it has to be. Several diagnostic post-processing steps applied to the CT volume (collectively termed “3D visualization”) must be repeated to generate an STL file that is then 3D printed. Multiple software packages are typically required before the STL file is electronically placed on a separate build-tray software platform. In 5 years or less, the inefficiency of medical modeling will be a historical footnote.

Current 3D printing publications are disparate. My group’s summary of the literature (submitted for publication in October 2014) attempted a comprehensive survey of the field stratified by organ section [1]. I personally apologize if your article was not included. However, those papers we did find and include spanned over 50 different journals.

*3D Printing in Medicine* is designed to provide a common platform peer-review platform. This forum is long overdue. The journal also addressed another missing piece: STL files are invited for submission and can be downloaded for free consumption by our readership. Those engaged in 3D printing are talented, and their creativity should be rewarded with development opportunities. *3D Printing in Medicine* invites not only clinical studies, but also “concept papers” that will motivate and connect physicians, industry, engineers, and scientists in general. These papers will benefit from peer review and serve as a platform for funding that will drive further innovations.

The journal will also address the question, “What defines a model that is clinically useful?” There are no 3D printing appropriateness criteria guidelines for a specific clinical scenario. Even the scenarios themselves are yet to be clearly defined. However, the challenge of clinical reimbursement will follow guidelines, and those guidelines in turn must be driven by peer-review studies that show that specific models are not only safe and efficacious, but also that they improve patient outcomes.

*3D Printing in Medicine* will promote literature standardization. Currently publications incompletely report methodology, limiting reproducibility and careful assessment of appropriateness. The journal will adopt a format for standardized enhance communication. A template format would include the following: printer type, materials, time to print (assuming the object was printed by itself), estimated cost of the materials, and potential overall cost to fabricate the model. Reporting should also include details regarding the print layer thickness and details regarding imaging if the model was created from DICOM images.

Precision remains critical for diagnoses and treatment. An early journal article addresses STL file precision to open a conversation among clinical modelers regarding best practice strategies [2]. Many clinical modelers are our trainees and academic junior staff members who have embraced 3D visualization. I welcome this talented, enthusiastic group to explore *3D Printing in Medicine* and translate their inventive spirit to the clinical and scientific communities, and, in the process, make meaningful contributions to improve healthcare.
